# Editorial

**Published:** 1881-04

**Authors:** 


### Editorial.

At the late banquet of the Alumni Association of Rush Medical College, it was expected that Dr. Allport would respond to the sentiment, "Dentistry and Dental Education, its Past, Present and Future as related to Medicine." The speakers on this occasion were however restricted in time to five minutes, and thus he was only able to give a brief synopsis of what he had intended to say, reserving his address in full for the transactions of the Association. We however anticipate this publication by presenting his address to our readers, in the present issue of the JOURNAL AND EXAMINER. That those who practice in the department of dentistry known as dental surgery, or the treatment of the natural teeth and their surroundings, should be medically educated and therefore regarded as special practitioners in medicine, can hardly be questioned. But that dental practitioners *not* medically educated should be admitted as medical practitioners, is scarcely debatable. The only door to the medical profession is through regularly constituted medical colleges; and we would suggest to our dental friends that the means for acquiring the highest scientific knowledge of their calling, should be found in these institutions. But it should be remembered that a full medical education has not been required or demanded of those about entering upon dental practice.

The suggestion that medical graduates should be better informed regarding this subject is worthy of consideration, and we do not see how the position taken by the doctor can be well controverted, for it is a fact, as said by the physician to whom he refers, that medical men "do not know much about the teeth." When a full medical education is demanded of those entering upon the practice of dental and oral surgery (and the sooner it is demanded the better) we have no doubt that medical colleges will prepare themselves to meet the demand, and instruct not only as to the diseases incident to that portion of the body and the science of their treatment, but provide such clinical teaching as will best qualify the student to put his knowledge into practice.

The suggestion made that medically educated men who practice dental and oral surgery should be received in the American Medical Association on an equal footing with the practitioners in any other department of medicine and have assigned to them a section on dental and oral surgery, claims attention. When the demand is made we do not see why it should not meet with a response. This department of practice should advance so as to stand among the important specialties of medicine.

---

AROMATIC TETRACHLORIDE OF CARBON.—Tetrachloride of Carbon was brought into notice as an anæsthetic and sedative by Dr. Protheroe Smith, in a series of papers which appeared in the *Lancet* of May and June, 1867. It is a colorless, exceedingly volatile liquid, having a delicate odor not unlike that of quince. From Dr. Protheroe Smith's papers it appears that when inhaled this body is very effective in removing the pains of labor and of dysmenorrhœa, and that it has been extensively and successfully employed for headache, toothache, neuralgia, lumbago, and rheumatic pains, applied externally on American leather cloth. Dr. Protheroe Smith has found it very efficacious in the mitigation and cure of hay-fever. He usually prescribes a very purified and perfumed compound manufactured by Corbin & Company which he calls Aromatic Tetrachloride of Carbon, and for which there is is considerable demand. "This new sedative, to which Dr. Protheroe Smith called attention in the *Lancet* about ten years ago, is prepared in a very agreeable form by Messrs. Corbyn. It has an odor like that of quince, and is said to have been useful in removing the pains of labor and dysmenorrhœa, in hay-fever and in neuralgia."—*Lancet*, July 13, 1878.

